# Editorial for the Special Issue: “Spatial Structure of Minerals”

**DOI:** 10.3390/molecules28207226

**Published:** 2023-10-23

**Authors:** Dun Wu, Guangqing Hu, Yuhang Gao

**Affiliations:** 1 Key Laboratory of Intelligent Underground Exploration, College of Civil Engineering, Anhui Jianzhu University, Hefei 230601, China; yhgaust@163.com; 2 School of Earth and Space Sciences, University of Science and Technology of China, Hefei 230026, China; 18855146880@163.com

## 1. Introduction

The spatial structure of minerals is a fundamental factor in determining the morphology, physical properties, and genesis of minerals [1,2,3,4]. Today, characterization techniques used to investigate various features of the spatial structure of minerals include X-ray diffraction (XRD), thermogravimetric analysis (TG), Fourier transform infrared spectroscopy (FTIR), Raman spectroscopy (Raman), and morphological observation techniques such as scanning electron microscopy (SEM) and atomic force microscopy (AFM) [5,6]. The characterization of the spatial structure of minerals can provide a scientific basis for explaining the genesis and evolution of minerals, as well as revealing geological phenomena [7,8,9].

In recent years, research activity on the interaction mechanism and environmental effects at the surface interface between minerals and environmental substances has been increasing [10,11]. In conjunction with the latest mineralogical research methods such as synchrotron radiation, in situ spectroscopy, adsorption modeling, and computational simulation, the surface interface processes and mechanisms of minerals have been explored at the atomic and molecular levels, as have the constraining mechanisms and the geochemical behaviors of environmental substances interacting with surface source minerals [12].

Thermal and kinetic studies on the spatial structure of minerals are also of great interest to the scientific community. Under high-pressure (i.e., high-temperature) conditions, corresponding patterns of change and intrinsic relationships in the chemical composition, internal structure, and physicochemical properties of minerals can be observed [13,14,15]. The study of high-pressure new minerals and the mechanism of mineral phase transition are of great significance to the development and utilization of mineral resources.

This Special Issue reflects the diversity of spatial structure studies related to minerals. We will provide a brief overview of the contents of this Special Issue in the following paragraphs. We would like to make it clear, however, that the purpose of this Editorial is not to elaborate extensively on each article, but to encourage readers to explore them.

## 2. An Overview of the Published Articles

Kang et al. (Contribution 1) [16] treated pyrite tailings slag using microbially induced carbonate precipitation (MICP) technology. The mineral composition and the spatial structure of the biocement samples were analyzed using X-ray diffraction (XRD) and Fourier transform infrared absorption spectroscopy (FTIR); the thermal stability and microstructure of the tailings slag were investigated via thermogravimetric analysis (TGA) and scanning electron microscopy (SEM). The results showed that biocementation of pyrite tailings using MICP technology can effectively reduce the permeability of the tailings. They further revealed the biochemical mechanism underlying biocementation of pyrite tailings formed via MICP and found that calcium carbonate precipitation was only induced after complex biochemical and physicochemical reactions. Through further investigations, the authors found that the generated calcium carbonate can also co-precipitate with heavy metal ions and that the carbonate precipitation on the surface of the tailings will further inhibit the tailings from oxidizing and prevent the diffusion of heavy metals. On the basis of forming a biocement barrier on the surface of the tailings sand, this study provides a theoretical basis for the study of the interactive mechanism between the surface interface of minerals and environmental substances and the environmental effects of this.

Luo et al. (Contribution 2) [17] observed four thermally altered coal-based graphite (TACG) samples using polarizing microscopy and Raman spectrometry and the Zhuji coal mine in the Huainan coal field as a case study. The results show that the degree of graphitization intensifies as the distance from the intrusion decreases, while the Raman wave peaks are shifted in the direction of a low wave number, the intensity of the G peaks (graphite) increases, the intensity of the D peaks (disorder) decreases, and the full width at maximum (FWHM) of the D peaks and the G peaks also decreases gradually. After a theoretical analysis of the spatial structure of TACG, the authors arrived at some interesting conclusions. They observed that a magma intrusion along the top plate of the coal seam reduced the degree of lattice defects during graphitization, and the areas of structural defects in the samples were attributed to primary structural defects. They also observed that the clay minerals in the coal seams were distributed in bands and demonstrated a symbiotic relationship between hydrothermal mineralization and coal and clay minerals, suggesting that sphalerite and pyrite are the products of the late stage of magmatic hydrothermal mineralization. In addition, this study demonstrated the intrinsic impact of geological pressure on the formation of coal seams, which provides an important reference for and a new perspective on the genesis and evolution of thermally metamorphosed coal-based graphite in the Huainan coal field.

Shi et al. (Contribution 3) [18] carried out a study on the activation of biotite-based polymers bound by sodium hydroxide to investigate the modification of clay using biotite-based polymers. The authors also assessed the effects of multiple factors on the unconfined compressive strength of the modified clay. The results showed that increasing the molar concentrations of the metakaolin-based geopolymer (MKG) and alkali activator increased the strength of the MKG, and the optimal experimental ratios were obtained, with the damage mode of the soil changing to brittle damage after treatment. The authors characterized the spatial structure of the modified clay via scanning electron microscopy (SEM) and X-ray diffraction (XRD), which showed that the binder not only acted as a bonding agent but also reacted with the clay to form a geopolymer, which produces gelling compounds that will bond the clay particles firmly together and increase their strength. This article also emphasizes that the treated soil has a denser structure, and that treatment enhances the mechanical properties of the soil. This study provides a possible application of metakaolin in soil improvement works.

The article by Hua et al. (Contribution 4) [19] describes how a new type of ceramite was prepared using iron tailings as the main raw material under a nitrogen atmosphere at 1150 °C. The ceramite was characterized and analyzed in detail via XRF, XRD, SEM-EDS, TGA, and specific surface area analysis. The results show that the prepared ceramite has a stable structure and is uniformly dense, contains a small number of particles, is high-strength, and exhibits good adsorption properties. In addition, the spatial structure of the pyroxene and tremolite in the ceramite resulted in an improved performance in terms of resistance to hydration. Further investigations revealed that silica, calcium oxide, and alumina are the main constituents of ceramite and suggested that mineral phases containing Al, Mg, or Ca are partially formed, with higher molecular weights, through complex chemical reactions. In this study, iron tailings were used as a material to fabricate lightweight high-strength ceramic stones with, which provides new possibilities for the reuse of iron tailing resources.

Zhao et al. (Contribution 5) [20] utilized uniaxial compression tests to analyze Pisha sandstone under a dry–wet cycle and to evaluate the changes in the mechanical properties of the specimens after the dry–wet cycle. The chemical composition and spatial structure of the specimens were analyzed via XRD and SEM. Results show that, on the one hand, a higher number of dry–wet cycles deteriorate the mechanical properties of the Pisha sandstone, resulting in a more fragile damage pattern (peeling damage). As the number of dry–wet cycles increases, the relative content of the clay minerals increases rapidly and then slowly, while the relative content of the primary minerals gradually decreases. On the other hand, it was found that the sandstone grains became rough and that a significant increase in porosity occurs. The authors also quantified the deterioration trend of the pore structure and put forward a predictive model for the correlation between uniaxial compressive strength and porosity. These results provide a reference for analyzing the evolution of Pisha sandstone slopes and predicting the geological phenomena of Pisha sandstone in its natural state.

## 3. Conclusions

This Special Issue introduces the latest research on the physical and chemical properties and modification mechanisms of pyrite tailings, the intrinsic structure and geological genesis of thermally metamorphosed coal-based graphite, the mechanical properties and spatial structure of geopolymer-modified clays, the characterization and utilization of the mineral phases of iron ore tailings, and the spatial structure and mechanical properties of Pisha sandstones under wet and dry cycling. This compilation of articles on the spatial structure of minerals covers a diverse range of research directions, reflected, for example, in the variety of research methods (such as the different techniques and equipment used to characterize minerals), the flexibility of combining macro- and micro-applications, and the range of media studied, such as rocks, industrial raw materials, and solid wastes. In addition, the results presented in this Special Issue can serve as a reference for scholars working in this research area. We invite such scholars to continue conducting further research on the topics discussed.
